# Patient Attitudes and Factors Associated with Willingness to Deprescribe Prescribed Medications in Western Saudi Arabia: A Cross-Sectional Study

**DOI:** 10.3390/healthcare14162511

**Published:** 2026-08-12

**Authors:** Mohammed S. Alharthi, Nehmat Ghaboura, Daniyah A. Almarghalani, Masi A. Almalki, Nasser M. Alorfi, Ahmed Alnemari, Ahmed Alshehri, Fahad T. Alsulami, Musaad M. Althobaiti

**Affiliations:** 1 Department of Clinical Pharmacy, College of Pharmacy, Taif University, Taif 21944, Saudi Arabia; a.alnemari.pharma@gmail.com (A.A.); a7med.alsheri@gmail.com (A.A.); f.alsulami@tu.edu.sa (F.T.A.); 2 Pharmacy Program, Department of Pharmacy Practice, Batterjee Medical College, Jeddah 21442, Saudi Arabia; pharmacy8.jed@bmc.edu.sa; 3 Department of Pharmacology and Toxicology, College of Pharmacy, Taif University, P.O. Box 11099, Taif 21944, Saudi Arabia; almarghalani@tu.edu.sa (D.A.A.); ma.malki@tu.edu.sa (M.A.A.); md.althobaiti@tu.edu.sa (M.M.A.); 4 Stroke Research Unit, Taif University, P.O. Box 11099, Taif 21944, Saudi Arabia; 5 Pharmacology and Toxicology Department, College of Pharmacy, Umm Al-Qura University, Makkah 21955, Saudi Arabia; nmorfi@uqu.edu.sa

**Keywords:** deprescribing, polypharmacy, medication therapy management, patient preferences, revised patients’ attitudes towards deprescribing questionnaire, Saudi Arabia

## Abstract

**Background:** Deprescribing is a supervised, patient-centred process for reducing or stopping medicines when their burden, harm, or lack of continuing indication outweighs expected benefits. This study assessed attitudes toward deprescribing, examined the exploratory psychometric performance of the revised Patients’ Attitudes Towards Deprescribing (rPATD) questionnaire, and identified factors associated with stated willingness to deprescribe among adults using prescribed medicines in western Saudi Arabia. **Methods:** An analytical cross-sectional survey was conducted from 15 February to 30 April 2025 in six community pharmacies and three outpatient clinics in Taif, Makkah, and Jeddah. Adults taking at least one prescribed medicine completed a paper-based Arabic rPATD questionnaire. Internal consistency, exploratory factor analysis, Spearman correlations, Mann–Whitney U tests, ordinal logistic regression, and a fully adjusted binary logistic regression sensitivity analysis were used. **Results:** Of 362 adults assessed, 320 were included in the final analysis. Overall, 254/320 (79.4%) agreed or strongly agreed that they would stop one or more medicines if their physician recommended it. Cronbach’s alpha ranged from 0.77 to 0.84, and McDonald’s omega ranged from 0.79 to 0.85. The four-factor exploration solution explained 61.4% of the variance. Greater involvement (adjusted odds ratio [aOR] 2.84, 95% confidence interval [CI] 1.98–4.06) and greater perceived medication inappropriateness (aOR 2.31, 95% CI 1.67–3.20) were associated with higher willingness, whereas concerns about stopping were associated with lower willingness (aOR 0.54, 95% CI 0.39–0.75). Medication burden was correlated with willingness but was not independently associated after adjustment (aOR 1.28, 95% CI 0.94–1.75; *p* = 0.112). **Conclusions:** Participants reported generally favourable stated willingness to deprescribe when advised by a physician. Involvement, perceived medication inappropriateness, and concerns about stopping were independently associated with willingness. These cross-sectional findings concern intention rather than actual deprescribing behaviour or clinical outcomes.

## 1. Introduction

The utilisation of medication becomes progressively intricate as populations age and multimorbidity gains wider prevalence. Many patients require long-term pharmacotherapy to prevent disease progression and reduce complications. This can lead to polypharmacy, commonly defined as the regular use of five or more medicines [1]. Polypharmacy may be clinically appropriate when each medicine has a clear indication; however, it becomes problematic when treatments are unnecessary, duplicated, poorly tolerated, or no longer aligned with current therapeutic goals. Such exposure is associated with adverse drug events, drug–drug interactions, reduced adherence, functional decline, hospitalisation, and an increased healthcare burden, particularly among older adults and people with chronic conditions [2]. Deprescribing is the planned and supervised process of reducing or discontinuing medicines when potential harms outweigh benefits, when treatment is no longer indicated, or when therapy is inconsistent with a patient’s preferences and goals [3]. It is not unsupervised medication withdrawal. Rather, it requires medication review, benefit–risk assessment, shared decision-making, an appropriate tapering or discontinuation plan, and follow-up monitoring [4,5,6]. Patients’ beliefs and preferences are central to deprescribing. Perceived medication necessity, concerns about withdrawal or symptom recurrence, trust in the physician, previous experiences, and the desired level of involvement can facilitate or hinder acceptance [7,8]. Clinician-level and organisational barriers also influence whether deprescribing discussions take place [9]. The Patients’ Attitudes Towards Deprescribing questionnaire and its revised version were developed to assess patients’ beliefs, experiences, and willingness to stop taking medicines [10,11]. A systematic review and meta-analysis of 29 studies involving 11,049 patients or caregivers found that most respondents were willing to consider deprescribing when recommended by a physician [12]. A multinational survey of older adults in 14 countries subsequently found substantial cross-national variation, suggesting that healthcare context, medication satisfaction, trust, and communication may shape attitudes [13]. Evidence from Arabic and Gulf populations remains limited. The Arabic rPATD was translated and psychometrically evaluated in Jordan [14]. Studies in Kuwait and Saudi Arabia have also reported generally favourable attitudes, although willingness varied by patient- and medication-related characteristics [15,16]. These findings support culturally situated research rather than the direct transfer of evidence from Western healthcare systems to Saudi practice. Deprescribing is most clinically consequential among older adults, people with multimorbidity, and those with polypharmacy. The present study, however, included adults taking at least one prescribed medication to examine attitudes across a broader population exposed to medication and to identify potential opportunities for earlier medication review. This broad eligibility criterion increases attitudinal coverage but requires cautious interpretation, as the sample was not restricted to patients at the highest clinical risk from polypharmacy. Accordingly, this study aimed to describe attitudes towards deprescribing, conduct an exploratory assessment of the rPATD domain structure and internal consistency, and identify factors associated with stated willingness to stop one or more prescribed medicines if recommended by a physician. The study also examined bivariate relationships among rPATD domains and assessed whether polypharmacy and perceived medication-related financial burden were associated with willingness to take medications.

## 2. Methods

### 2.1. Study Design and Setting

An analytical cross-sectional survey design was employed because the exposures of patient attitudes, sociodemographic characteristics, and medication-related factors, and the outcome, stated willingness to deprescribe, were measured at a single point during the same study period. This methodological approach is suitable for describing the distribution of attitudes and estimating prevailing associations; however, it does not permit the establishment of temporal sequences or causal relationships. Data collection was conducted from 15 February to 30 April 2025 across six community pharmacies and three general outpatient clinics in Taif, Makkah, and Jeddah within the Makkah Region of western Saudi Arabia. Participants independently completed a paper-based Arabic questionnaire. In this context, “self-administered” indicates that participants read the items and recorded their own responses. Trained research assistants explained the procedures for obtaining consent and, when necessary, completed the questionnaire, but they did not interpret questionnaire items or select responses on participants’ behalf.

### 2.2. Study Population, Sample Size, Recruitment, and Participant Flow

Eligible participants were adults aged 18 years or older who were currently taking at least one prescribed medicine, could provide informed consent, could read Arabic, and could complete the questionnaire independently. Exclusion criteria were current use of prescribed medication, inability to provide informed consent, inability to complete the questionnaire without a proxy, acute illness requiring immediate care, prior participation, or more than 20% missing data on the questionnaire. Polypharmacy was defined a priori as the regular use of five or more medicines [1]. The broad inclusion criterion was selected to capture deprescribing attitudes across the spectrum of prescribed medicine use rather than only among older adults or people with polypharmacy. This approach permits assessment of willingness before medication burden becomes severe, but it limits direct generalisation to geriatric, multimorbid, and clinically complex polypharmacy populations. The minimum sample size was calculated using Cochran’s formula for a single proportion, assuming a conservative expected prevalence of willingness of 50%, a 95% confidence level, and an absolute precision of 5.6%. The calculation yielded a minimum of 306 participants. The recruitment target was 340 after allowing approximately 10% for non-returned or incomplete questionnaires. The final sample of 320 exceeded the minimum, providing 16 participants per analysed rPATD domain item for exploratory factor analysis and 45.7 participants per parameter in the seven-variable ordinal regression model. Participants were recruited by convenience sampling during predefined recruitment sessions. Trained research assistants approached potentially eligible adults in pharmacy dispensing areas and outpatient waiting areas, screened eligibility using a brief checklist, and provided the study information sheet. Recruitment was consecutive within each session, and no financial or other incentive was offered. Written informed consent was obtained before the questionnaire was issued. Of 362 adults assessed for eligibility, 343 met the inclusion criteria. Nineteen were ineligible because they were not taking a currently prescribed medication (*n* = 11) or could not complete the questionnaire independently (*n* = 8), and 9 eligible adults declined participation. Of the 334 participants who consented and received a questionnaire, 328 returned it; 6 did not return it and contributed no data. Eight returned questionnaires were excluded during data screening because more than 20% of responses were missing (*n* = 6) or the record was a duplicate (*n* = 2). The final analytic sample was 320. The response rate among eligible adults was 95.6% (328/343), and the analytic completion rate among consenting participants was 95.8% (320/334). No partial data from excluded or non-returning participants was included.

### 2.3. Questionnaire and Measures

Patients’ attitudes were measured using the 22-item Arabic self-report version of the revised Patients’ Attitudes Towards Deprescribing questionnaire (rPATD), originally developed by Reeve et al. [10] and translated and psychometrically evaluated in Arabic by Nusair et al. [14]. The self-report version was used because all participants answered about their own prescribed medicines. Written permission for non-commercial academic use of the Arabic version was obtained before data collection. The instrument remains copyrighted by its developers; therefore, the full item wording is not reproduced in this article. The instrument contains 20 domain items and two global items. The four five-item domains are medication burden, perceived medication inappropriateness (the original “appropriateness” factor, scored so that higher values indicate greater belief that one or more medicines may be unnecessary or unsuitable), concerns about stopping, and involvement in medication-related decision-making. The global items assess satisfaction with current medicines and willingness to stop one or more regular medicines if the participant’s physician states that this is possible. The willingness item was the primary outcome. All items were rated on a five-point Likert scale from 1 (strongly disagree) to 5 (strongly agree). The Arabic scoring key did not require reverse coding of the 20 domain items. A domain score was calculated as the mean of its five items when at least four items had been answered; otherwise, the domain score was treated as missing. Higher scores represent greater burden, greater perceived medication inappropriateness or reduced necessity, greater concerns about stopping, and greater involvement, respectively. The primary outcome retained all five ordered willingness categories. For the binary sensitivity analysis, “agree” and “strongly agree” were classified as willing, whereas “strongly disagree”, “disagree”, and “neither agree nor disagree” were classified as not willing or uncertain. Other variables were age, sex, the exact number of regularly prescribed medicines, polypharmacy status, self-reported medication-related side effects, and perceived medication-related financial burden.

### 2.4. Cultural Adaptation and Pilot Testing

The validated Arabic rPATD underwent local review before field administration. A panel comprising two clinical pharmacists, one family physician, and one researcher with expertise in survey methodology assessed semantic clarity and contextual suitability without altering the construct content. Cognitive debriefing with 15 adults confirmed that the instructions and response options were understood. A separate pilot study with 25 adults found a median completion time of 8 min, no item-level missingness exceeding 4%, and domain Cronbach’s alpha values ranging from 0.75 to 0.83. No substantive item changes were required. Cognitive debriefing and pilot participants were excluded from the main recruitment frame, and the final sample was 320.

### 2.5. Data Management

Completed paper questionnaires were double-entered independently into Microsoft Excel version 2607 (Build 20228.20158; Microsoft Corporation, Redmond, WA, USA), and discrepancies were resolved by reference to the original form. Analyses were conducted using IBM SPSS Statistics, version 27. The dataset was screened for consent, eligibility, duplicate records, completeness, internal consistency, and implausible values. Multiple responses to a single-choice item were treated as missing. A questionnaire was excluded if more than 20% of items were missing. For a domain with one missing item, the score was calculated from the four completed items; domains with two or more missing items were set to missing. No included participant had missing outcome or covariate data after screening, so all reported descriptive, correlation, group-comparison, and regression analyses used complete cases (*N* = 320). No statistical imputation was performed.

### 2.6. Statistical Analysis

Categorical variables were summarised as frequencies and percentages. Likert items and domain scores were reported using means and standard deviations for comparison with prior rPATD studies, and medians with interquartile ranges to reflect their ordinal distributions. The distribution of all five willingness-response categories, as well as the combined percentage of respondents who agreed or strongly agreed, was reported. Internal consistency was assessed using Cronbach’s alpha, McDonald’s omega, and corrected item–total correlations. Suitability for exploratory factor analysis (EFA) was assessed using the Kaiser–Meyer–Olkin measure and Bartlett’s test of sphericity. Principal-axis factoring with Promax oblique rotation (κ = 4) was used because the domains were expected to correlate. Factor retention was based on parallel analysis, the scree plot, eigenvalues greater than 1, and theoretical interpretability. Pattern coefficients of at least 0.40 were considered salient, and cross-loadings of at least 0.30 were examined. Eigenvalues, variance explained, communalities, and the full item-level pattern matrix were reported. Because confirmatory factor analysis was not performed, the analysis was interpreted as exploratory support rather than definitive confirmation of construct validity. Spearman’s rank correlation coefficient quantified bivariate associations among rPATD domains and willingness. Mann–Whitney U tests compared willingness by polypharmacy and perceived financial burden. Results included group medians and interquartile ranges, the U statistics, two-sided *p*-values, and rank-biserial correlations with 95% confidence intervals. Absolute rank-biserial correlations of approximately 0.10, 0.30, and 0.50 were interpreted as small, moderate, and large, respectively. Ordinal logistic regression examined factors independently associated with higher willingness across the five ordered response categories. The four rPATD domain scores were included a priori because they represent the instrument’s conceptual dimensions. Exact medication counts, age, and sex were included as clinically plausible covariates identified before analysis. Medication count was entered continuously, so its adjusted odds ratio represents one additional regular medicine. Age was coded as 50 years or older versus younger than 50 years because this cut-point corresponded to the prespecified age bands and maintained adequate cell sizes; sex was coded as female versus male. Polypharmacy was not entered with medication count because the two variables encode the same exposure, and side effects and financial burden were retained as secondary unadjusted comparisons to avoid over-parameterisation and potential mediation through the attitude domains. Results are presented as adjusted odds ratios (aORs) with 95% confidence intervals. A binary logistic regression sensitivity analysis used the dichotomised willingness outcome and the same seven covariates as in the ordinal model. For the ordinal model, the proportional-odds assumption was evaluated using the parallel-lines test; overall likelihood ratio significance, Pearson and deviance goodness-of-fit tests, Nagelkerke pseudo-R^2^, multicollinearity, and influential observations were also examined. Variance inflation factors below 5 and tolerance above 0.20 were considered acceptable. Linearity of the continuous terms on the log-odds scale was checked using score-by-log-score interaction terms. For the binary model, calibration was assessed using the Hosmer–Lemeshow test and discrimination using the area under the receiver operating characteristic curve. All tests were two-sided, and *p* < 0.05 was considered statistically significant.

### 2.7. Ethical Approval

Ethical approval was obtained from Taif University’s Scientific Research Ethics Committee, accredited by the National Committee for Bioethics under registration number HAPO-02-T-105. Approval was granted under application number 45–146 on 29 January 2025. Participation was voluntary, responses were treated confidentially, and informed consent was obtained before completing the questionnaire.

## 3. Results

### 3.1. Participant Flow and Characteristics

**Figure 1:**
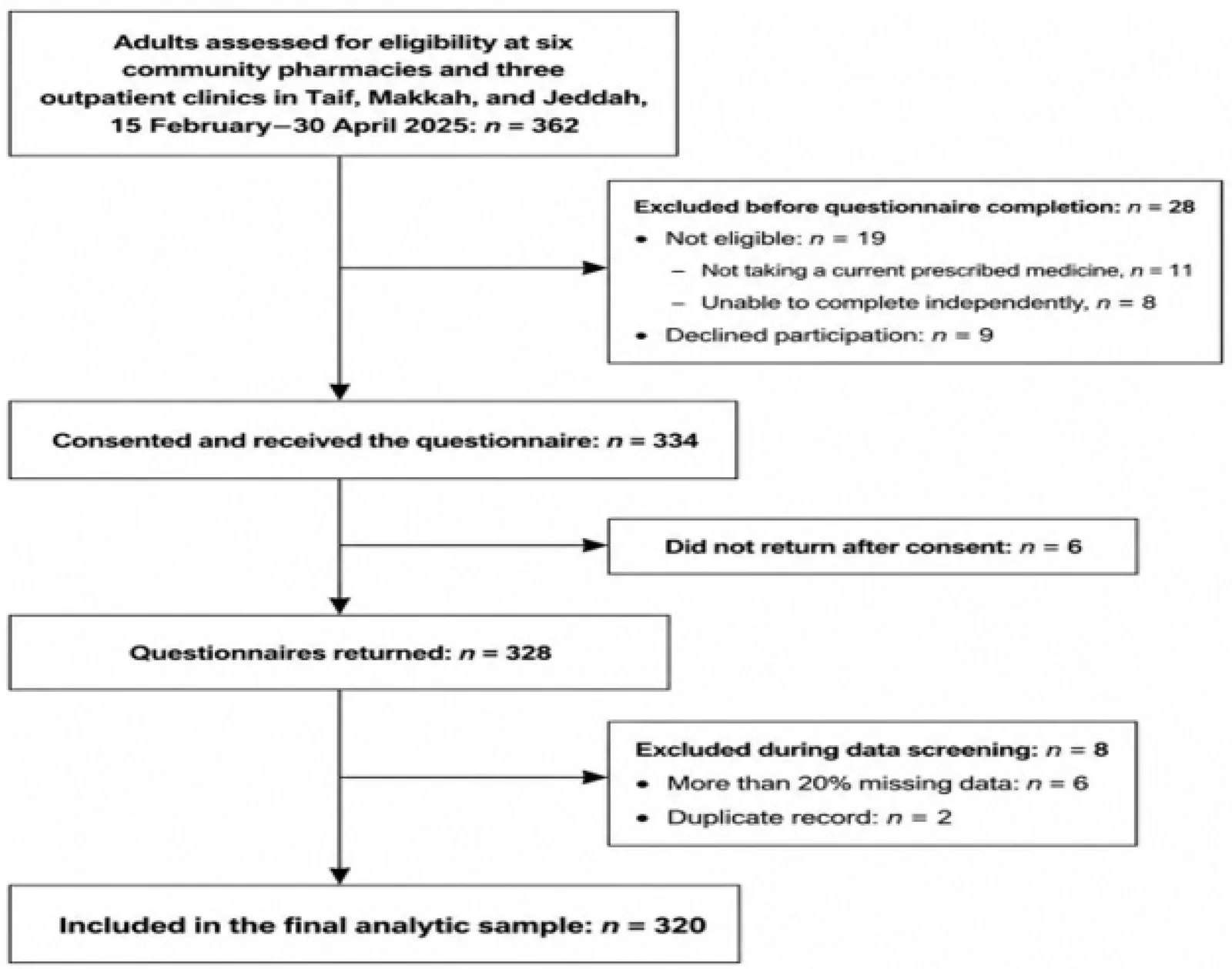
Participant recruitment and analytic flow.

Figure 1 presents participant disposition. Of 362 adults assessed, 334 consented and received the questionnaire, 328 returned it, and eight returned questionnaires were excluded. The final analytic sample comprised 320 participants. Of these, 208 (65.0%) were female, 250 (78.1%) were younger than 50 years, and 96 (30.0%) were taking five or more regular medicines. Medication-related side effects were reported by 182 participants (56.9%), and 174 (54.4%) reported a medication-related financial burden (Table 1).

### 3.2. rPATD Descriptive Statistics and Reliability

Responses to the willingness item were categorised as follows: strongly disagree, 1 (0.3%); disagree, 12 (3.8%); neither agree nor disagree, 53 (16.6%); agree, 148 (46.2%); and strongly agree, 106 (33.1%). Overall, 254 of 320 participants (79.4%) expressed agreement or strong agreement with the idea of ceasing one or more regular medications if their physician indicated it was feasible. The mean willingness score was 4.08 (SD 0.82), with a median of 4.00 (interquartile range 4.00–5.00). The involvement domain had the highest mean score, 3.95 (SD = 0.66). Cronbach’s alpha coefficients ranged from 0.77 for concerns about cessation to 0.84 for involvement, while McDonald’s omega coefficients ranged from 0.79 to 0.85 (see Table 2). Corrected item–total correlations ranged from 0.52 to 0.68 for burden, 0.47 to 0.63 for perceived inappropriateness, 0.44 to 0.60 for concerns, and 0.55 to 0.71 for involvement.

### 3.3. Exploratory Factor Analysis

The Kaiser–Meyer–Olkin measure was 0.889, and Bartlett’s test of sphericity was significant (χ^2^ (190) = 2864.7, *p* < 0.001), supporting factorability. Parallel analysis and the scree plot supported retention of four factors. Their eigenvalues were 6.02, 2.72, 1.86, and 1.67, explaining 30.1%, 13.6%, 9.3%, and 8.4% of the variance, respectively; cumulative explained variance was 61.4%. Communalities ranged from 0.44 to 0.67. All 20 items loaded at least 0.61 on their intended factor; no cross-loading exceeded 0.30; and the complete pattern matrix is presented in Table 3. Because the analysis was exploratory and confirmatory factor analysis was not performed in an independent sample, these findings provide preliminary support for the proposed four-domain structure rather than definitive confirmation of construct validity.

### 3.4. Bivariate Associations Among rPATD Domains and Willingness

Willingness was positively correlated with medication burden (r_s_ = 0.46), perceived medication inappropriateness (r_s_ = 0.50), and involvement (r_s_ = 0.53), and negatively correlated with concerns about stopping (r_s_ = −0.47); all *p* < 0.01. Correlations among the four domain scores ranged from 0.21 to 0.42 in absolute magnitude (Table 4). Involvement, rather than burden, had the largest bivariate correlation with willingness.

### 3.5. Group Comparisons

Among participants with polypharmacy, the median willingness was 5.00 (IQR 4.00–5.00), compared with 4.00 (IQR 3.00–5.00) among those without polypharmacy (U = 15,268, *p* < 0.001). Among those reporting medication-related financial burden, the median willingness was 5.00 (IQR 4.00–5.00), compared with 4.00 (IQR 3.00–4.00) among those without financial burden (U = 18,418, *p* < 0.001). The rank-biserial effect sizes were moderate to large (Table 5).

### 3.6. Ordinal Logistic Regression

The ordinal model included all 320 complete cases. Greater involvement (aOR 2.84, 95% CI 1.98–4.06; *p* < 0.001) and greater perceived medication inappropriateness (aOR 2.31, 95% CI 1.67–3.20; *p* < 0.001) were associated with higher willingness. Concerns about stopping were associated with lower willingness (aOR 0.54, 95% CI 0.39–0.75; *p* < 0.001). Each additional regular medicine was associated with higher willingness (aOR 1.19, 95% CI 1.02–1.39; *p* = 0.027). Participants aged 50 years or older had higher odds than those younger than 50 years (aOR 1.72, 95% CI 1.05–2.82; *p* = 0.031). Female sex was not associated with willingness (*p* = 0.381). Medication burden was correlated with willingness in the bivariate analysis (r_s_ = 0.46, *p* < 0.01) but did not retain an independent association after simultaneous adjustment for the other rPATD domains and covariates (aOR 1.28, 95% CI 0.94–1.75; *p* = 0.112). This attenuation is consistent with shared variance among burden, medication count, perceived inappropriateness, concerns, and involvement, rather than an independent effect of burden after adjustment. The parallel-lines test was non-significant, χ^2^ (21) = 24.18, *p* = 0.286, supporting the proportional-odds assumption. The overall model was significant, with a likelihood-ratio χ^2^ (7) = 164.52, *p* < 0.001, and Nagelkerke pseudo-R^2^ = 0.438. Pearson and deviance goodness-of-fit tests were non-significant (*p* = 0.418 and *p* = 0.691, respectively). Variance inflation factors ranged from 1.11 to 1.78, and tolerance ranged from 0.56 to 0.90. Score-by-log-score terms were non-significant (all *p* > 0.10), and no influential observation materially changed the estimates. The full ordinal logistic regression results are presented in Table 6.

### 3.7. Binary Logistic-Regression Sensitivity Analysis

In the fully adjusted binary sensitivity model, involvement (aOR 3.12, 95% CI 2.05–4.75; *p* < 0.001), perceived medication inappropriateness (aOR 2.54, 95% CI 1.78–3.63; *p* < 0.001), and concerns about stopping (aOR 0.49, 95% CI 0.34–0.71; *p* < 0.001) remained associated with willingness in the same direction as in the ordinal model. Medication counts also remained associated (aOR 1.16, 95% CI 1.01–1.34; *p* = 0.040), whereas burden, age, and sex were not statistically significant (Table 7).

## 4. Discussion

This cross-sectional survey found generally favourable stated willingness to stop one or more medicines when a physician recommended doing so. Greater involvement in medication-related decisions and greater perceived medication inappropriateness were independently associated with higher willingness, whereas concerns about stopping were linked to lower willingness. Medication count and age 50 years or older also showed positive adjusted associations. Medication burden was related to willingness in the bivariate analysis but was not independently associated after adjustment. The favourable willingness observed is consistent with international evidence showing that many patients will consider deprescribing when it is recommended by a trusted physician [12,13,17,18]. Nevertheless, the outcome was a stated intention in response to a questionnaire item. It should not be equated with the actual acceptance of a specific recommendation, successful discontinuation of medication, sustained adherence to a tapering plan, symptom stability, safety, or clinical benefit. Longitudinal and interventional studies are required to determine how stated willingness translates into behaviour. Involvement showed the largest adjusted association with willingness. This accords with studies from Singapore and Europe in which patients and caregivers valued participation, explanation, and collaborative medication review [19,20]. Shared decision- making may increase acceptability by enabling patients to understand the rationale for deprescribing, express treatment priorities, and agree on monitoring or restart plans [6]. The present design cannot establish that increased involvement leads to greater acceptance, but it identifies involvement as a relevant construct for future intervention testing. The positive association for the appropriateness- related factor becomes conceptually coherent when its scoring direction is made explicit. Higher scores in this study indicate greater perceived medication inappropriateness or reduced necessity, rather than a stronger belief that current medicines are appropriate. Patients who believed that one or more medicines might no longer be necessary, effective, or suitable were more willing to consider stopping them. This interpretation is consistent with medication- specific research showing that beliefs about necessity and suitability influence willingness [21,22]. Concerns about stopping were inversely associated with willingness. Fear of symptom recurrence, withdrawal effects, disease exacerbation, or loss of future benefit can make patients reluctant to discontinue long-standing therapy [7,23,24]. These concerns should be elicited rather than dismissed. Deprescribing conversations may be more acceptable when they include a clear clinical rationale, gradual tapering when indicated, explicit monitoring, safety-netting, and an agreed option to reconsider or restart treatment when clinically appropriate. Medication burden illustrates the distinction between bivariate and adjusted associations. Burden correlated with willingness (r_s_ = 0.46), although it was not the strongest bivariate correlate; involvement had the largest correlation (r_s_ = 0.53). After the four domains, medication count, age, and sex were entered simultaneously, the burden estimate was attenuated, and its confidence interval included the null. The domain correlations and VIFs of 1.11–1.78 do not indicate problematic multicollinearity, but they show sufficient conceptual overlap for the burden’s unique adjusted contribution to be smaller than its unadjusted relationship. Burden should therefore not be described as an independent factor in this model. Participants with polypharmacy and those reporting financial burden had a greater willingness in unadjusted comparisons. People managing more medicines may experience greater regimen complexity, adverse effects, inconvenience, or medication fatigue [17,25]. Financial strain may also prompt a reassessment of the value of treatment. However, deprescribing decisions must remain clinically led and should not result in discontinuation of beneficial therapy solely because of cost. Because only 30% of the sample met the polypharmacy definition and most participants were younger than 50 years, these findings should not be generalised uncritically to older adults with multimorbidity or complex, potentially inappropriate medicine use. The internal consistency estimates and exploration factor pattern were consistent with a four-domain structure. The Arabic rPATD has previously demonstrated acceptable psychometric properties [14], and the present analysis extends exploratory evidence to a Saudi sample. The use of parallel analysis, full cross-loading reporting, omega coefficients, and item–total correlations strengthens the assessment. However, the findings do not constitute definitive validation. Confirmatory factor analysis in an independent Saudi sample, assessment of measurement invariance across age and sex, and test–retest reliability would provide stronger evidence. Clinically, the findings support asking patients about their preferences for involvement, beliefs that medicines may no longer be necessary, and concerns about stopping during structured medication review. Pharmacists and physicians may use these domains to guide discussion, but the rPATD should not be used as a stand-alone decision rule. Actual deprescribing requires medicine-specific assessment of indication, expected benefit, current harm, patient goals, tapering requirements, and follow-up. The observed associations generate hypotheses for patient-centred interventions rather than evidence that a particular intervention is effective or safe.

## 5. Strengths and Limitations

Strengths include the use of a recognised deprescribing attitude instrument, assessment of several conceptually distinct domains, explicit reporting of the full willingness distribution, exploratory psychometric analysis with multiple reliability indices, and the use of both ordinal and binary multivariable models. The ordinal analysis retained information across all five outcome categories, while the fully adjusted binary sensitivity analysis demonstrated consistent directions of the principal associations.

Several limitations should be considered. The cross-sectional design precludes temporal and causal inference. Convenience sampling from community pharmacies and general outpatient clinics in Taif, Makkah, and Jeddah may have produced selection bias, despite a high response rate. The sample was predominantly female, younger than 50 years, and not predominantly composed of people with polypharmacy; generalisability to older adults, people with multimorbidity, and patients with clinically complex medication regimens is therefore limited. Self-reported medication use, side effects, financial burden, and willingness may also be affected by recall and social desirability bias. The study measured stated willingness rather than actual deprescribing acceptance, implementation, persistence, withdrawal events, symptom recurrence, or health outcomes. Important potential confounders and contextual variables were not collected, including comorbidities, chronic disease status, medicine classes and treatment duration, potentially inappropriate medicines, education, income, insurance status, health literacy, adherence, trust in physicians, and previous deprescribing experience. Residual confounding is therefore likely. The use of the older-adult self-report rPATD across a broad adult age range and the absence of confirmatory factor analysis also require cautious interpretation of the psychometric findings.

## 6. Conclusions

Adults in this western Saudi sample reported generally favourable stated willingness to stop one or more prescribed medicines when a physician recommended doing so. Greater involvement in medication-related decisions and greater perceived medication inappropriateness were associated with higher willingness, whereas concerns about stopping were associated with lower willingness. Medication burden was correlated with willingness but was not independently associated after adjustment. These results may inform future medication review and shared decision-making research, but they do not establish causal effects, actual deprescribing behaviour, safety, or clinical benefit.

## Figures and Tables

**Table 1:** Sociodemographic and Clinical Characteristics of Participants (*N* = 320).

Variable	Category	*n*	%
Gender	Male	112	35.0
	Female	208	65.0
Age group, years	18–29	124	38.8
	30–49	126	39.4
	≥50	70	21.8
Number of regular medications	1–2	96	30.0
	3–4	128	40.0
	≥5	96	30.0
Polypharmacy (≥5 medications)	Yes	96	30.0
	No	224	70.0
Experienced medication-related side effects	Yes	182	56.9
	No	138	43.1
Perceived medication-related financial burden	Yes	174	54.4
	No	146	45.6

Percentages were calculated using *N* = 320. Display rounding was standardised so that mutually exclusive categories sum to 100.0%.

**Table 2:** Descriptive Statistics and Internal Consistency of rPATD Domains.

Domain/Measure	Items, *n*	Mean	SD	Median (IQR)	Cronbach’s α (McDonald’s ω)
Medication burden	5	3.62	0.78	3.60 (3.00–4.20)	0.81 (0.82)
Perceived medication inappropriateness	5	3.28	0.71	3.20 (2.80–3.80)	0.79 (0.80)
Concerns about stopping	5	3.11	0.74	3.00 (2.60–3.60)	0.77 (0.79)
Involvement	5	3.95	0.66	4.00 (3.60–4.40)	0.84 (0.85)
Willingness to deprescribe (single item)	1	4.08	0.82	4.00 (4.00–5.00)	—

Higher scores on perceived medication inappropriateness indicate a stronger belief that one or more current medicines may be unnecessary or unsuitable. IQR, interquartile range; rPATD, revised Patients’ Attitudes Towards Deprescribing questionnaire; SD, standard deviation.

**Table 3:** Exploratory Factor Analysis Pattern Matrix and Communalities.

Abbreviated Item Descriptor	Burden	Perceived Inappropriateness	Concerns	Involvement	Communality
B1: Perceived number of medicines is large	0.74	0.12	−0.05	0.08	0.61
B2: Medicines are experienced as burdensome	0.79	0.08	0.02	0.05	0.66
B3: Daily medicine-taking is inconvenient	0.68	0.15	0.11	−0.04	0.52
B4: Medicines create a cost burden	0.61	0.09	0.14	0.03	0.44
B5: Desire to reduce the medicine load	0.72	0.18	−0.06	0.10	0.58
PI1: Medicine may no longer be needed	0.11	0.73	−0.08	0.12	0.59
PI2: Medicine may no longer be effective	0.16	0.69	0.04	0.09	0.54
PI3: Medicine may be causing adverse effects	0.20	0.65	0.10	0.04	0.51
PI4: Openness to a supervised trial of stopping	0.08	0.71	−0.12	0.15	0.57
PI5: A current medicine may be unsuitable	0.14	0.76	−0.05	0.11	0.63
C1: Reluctance to stop long-term medicine	0.06	−0.09	0.70	−0.10	0.53
C2: Fear of losing future treatment benefit	0.12	0.03	0.74	−0.08	0.58
C3: Stress when medicines are changed	0.17	−0.04	0.66	−0.14	0.50
C4: Fear that the condition will worsen	0.09	−0.08	0.72	−0.05	0.55
C5: Preference to continue familiar medicines	0.04	−0.11	0.63	−0.18	0.45
I1: Understanding the reason for each medicine	0.05	0.11	−0.07	0.76	0.61
I2: Knowledge of the current medicine list	0.09	0.08	−0.03	0.71	0.54
I3: Desire to participate in medicine decisions	−0.02	0.15	−0.10	0.79	0.67
I4: Asking questions when information is unclear	0.06	0.12	−0.09	0.74	0.59
I5: Confidence discussing medicines with a physician	0.10	0.18	−0.14	0.68	0.53

Extraction method: principal-axis factoring. Rotation: Promax with κ = 4. Factor-retention criteria: parallel analysis, scree-plot inspection, eigenvalues greater than 1, and theoretical interpretability. KMO = 0.889; Bartlett’s test, χ^2^(190) = 2864.7, *p* < 0.001. Pattern coefficients are reported; loadings with absolute values below 0.30 are shown to indicate the absence of substantial cross-loadings. Item descriptors are abbreviated paraphrases due to the instrument’s copyright.

**Table 4:** Spearman Correlation Matrix for rPATD Domains and Willingness (*N* = 320).

Variable	Burden	Perceived Inappropriateness	Concerns	Involvement	Willingness
Burden	—	0.41 **	−0.36 **	0.30 **	0.46 **
Perceived inappropriateness	0.41 **	—	−0.28 **	0.42 **	0.50 **
Concerns about stopping	−0.36 **	−0.28 **	—	−0.21 **	−0.47 **
Involvement	0.30 **	0.42 **	−0.21 **	—	0.53 **
Willingness	0.46 **	0.50 **	−0.47 **	0.53 **	—

Values are Spearman rank correlation coefficients (r_s_); *N* = 320. ** *p* < 0.01. Higher perceived-inappropriateness scores indicate greater belief that one or more medicines may be unnecessary or unsuitable.

**Table 5:** Group Comparisons for Willingness to Deprescribe.

Comparison	Group Medians (IQR)	Mann–Whitney U	*p*	Effect Size *
Polypharmacy: Yes (*n* = 96) vs. No (*n* = 224)	Yes: 5.00 (4.00–5.00) No: 4.00 (3.00–5.00)	15,268	<0.001	rrb = 0.42 (95% CI 0.32–0.51)
Financial burden: Yes (*n* = 174) vs. No (*n* = 146)	Yes: 5.00 (4.00–5.00) No: 4.00 (3.00–4.00)	18,418	<0.001	rrb = 0.45 (95% CI 0.36–0.54)

*: Effect size is the rank-biserial correlation, rrb = 2U/(n1n2) − 1, with positive values indicating greater willingness in the first-listed group. IQR, interquartile range.

**Table 6:** Ordinal Logistic Regression of Factors Associated with Willingness to Deprescribe.

Factor	Adjusted OR	95% CI	*p*
Involvement	2.84	1.98–4.06	<0.001
Perceived medication inappropriateness	2.31	1.67–3.20	<0.001
Concerns about stopping	0.54	0.39–0.75	<0.001
Medication burden	1.28	0.94–1.75	0.112
Number of medications *	1.19	1.02–1.39	0.027
Age ≥ 50 years †	1.72	1.05–2.82	0.031
Female sex ‡	1.21	0.79–1.85	0.381

OR: odds ratio; CI: confidence interval. *: OR per additional regular medication; †: age ≥50 years compared with <50 years; ‡: female compared with male.

**Table 7:** Fully Adjusted Binary Logistic-Regression Sensitivity Analysis.

Factor	Adjusted OR	95% CI	*p*
Involvement	3.12	2.05–4.75	<0.001
Perceived medication inappropriateness	2.54	1.78–3.63	<0.001
Concerns about stopping	0.49	0.34–0.71	<0.001
Medication burden	1.17	0.81–1.69	0.403
Number of medications	1.16	1.01–1.34	0.040
Age ≥ 50 years	1.58	0.88–2.84	0.126
Female sex	1.14	0.68–1.92	0.621

Model *N* = 320. Outcome: agree/strongly agree versus all other responses. The same seven covariates used in the ordinal model were retained. Overall likelihood-ratio χ^2^(7) = 126.31, *p* < 0.001; Nagelkerke R^2^ = 0.452; Hosmer–Lemeshow χ^2^(8) = 6.84, *p* = 0.554; area under the receiver-operating-characteristic curve = 0.866 (95% CI 0.824–0.909); overall classification accuracy = 82.8%. The medication-count OR is per one additional regular medicine; reference groups were age < 50 years and male sex.

## Data Availability

The data presented in this study are available on request from the corresponding author due to privacy and ethical restrictions related to participant confidentiality and informed consent.
